# The causal role between phasic midbrain dopamine signals and learning

**DOI:** 10.3389/fnbeh.2014.00139

**Published:** 2014-04-25

**Authors:** Luca Aquili

**Affiliations:** Department of Psychology, Sunway UniversityBandar Sunway, Petaling Jaya, Malaysia

**Keywords:** dopamine, reward prediction error, electrophysiology, errors, behavioral flexibility

Reinforcement learning occurs when organisms adapt behavior on the basis of associations with reward and punishment.Reinforcement learning is a useful algorithm because it is unsupervised, relying on trial-and-error learning under conditions in which the optimal solution is unknown. Recent neural network models of reinforcement learning are based on the neurophysiology of the rat, monkey, and human dopamine systems (Montague et al., 1996; Dayan and Balleine, 2002; Schultz, 2002; Montague et al., 2004; Pan et al., 2008). The main finding of this research is that the dopamine system appears to minimize errors in the prediction of reward through a process called *temporal difference learning*. As predicted by the temporal difference learning models, dopamine neurons respond during the early stages of classical and operant conditioning with a burst of action potentials (a phasic-like response) after reward presentation (Schultz, 1998; O'Doherty et al., 2006). However, after repeated pairings of a given stimulus and reinforcement, the dopamine neurons respond to the onset of the stimulus, be it a conditioned stimulus or a cue that triggers a stereotyped action that results in reward (Mirenowicz and Schultz, 1994). After an association has been formed between the stimulus and reinforcement, dopamine ceases responding to the reinforcer itself (Schultz et al., 1997).

Based on these neurophysiological data, reinforcement learning models have proposed that the role of the midbrain phasic DA neurons is to act as a teaching signal which adjusts reward prediction errors and broadcasts such information to upstream cell populations involved in reward learning such as the nucleus accumbens (NAc) (Joel et al., 2002; Wassum et al., 2013). More recently, a number of computational studies have added another layer of complexity to their models by incorporating the idea of incentive motivation as a way to better capture the role of dopamine in reward learning (McClure et al., 2003; Niv, 2007; Zhang et al., 2009; Morita et al., 2013). This has largely been based on findings from lesion and pharmacological studies whereby it has been hypothesized that dopamine neurons respond to conditioned stimuli by invigorating instrumental actions that lead to the obtainment of rewards (Berridge et al., 2009; Wassum et al., 2011).

In the meantime, a number of authors have suggested that because midbrain dopamine neurons also respond to aversive and salient stimuli by phasic DA activations (Matsumoto and Hikosaka, 2009; Cohen et al., 2012; Ilango et al., 2012; Tan et al., 2012; Brooks and Berns, 2013; Fiorillo et al., 2013), that their role in encoding reward prediction errors may be more limited than first envisaged (Horvitz et al., 1997; Redgrave and Gurney, 2006; Redgrave et al., 2008; May et al., 2009; Thirkettle et al., 2013). The scope of this Opinion article, however, is not to assess the validity of such claims.

On the contrary, the aim of this article is to focus on one area of research that has received relatively little attention, namely, how the phasic DA signal may be causally related to action selection, goal-directed behavior, and behavioral flexibility. This is partially because the vast majority of studies which have explored whether DA neurons may encode more than reward prediction errors (e.g., including measures related to behavioral flexibility such as reward value, reward probability, choice behavior, discounting of delayed rewards) (Fiorillo et al., 2003, 2008; Morris et al., 2006; Roesch et al., 2007; Takahashi et al., 2009; Bromberg-Martin et al., 2010a,b; Nomoto et al., 2010), have been based upon electrophysiological data, which by their very nature can only support a correlation between neuronal activation and inhibition with behavior but cannot establish causation. This has been acknowledged by a statement from Wolfram Schultz who declared that “although the prediction error response of dopamine neurons would make a good teaching signal, the bulk of the available data are correlational” (Schultz, 2010). Therefore, to establish causation we will look at a number of recent studies that have used primarily, optogenetic, voltammetry and pharmacological interventions and that may provide an answer to this question.

With the recent introduction of optogenetics, for example, it has been possible to perturb neural activity at millisecond timescales and directly relate this manipulation to an array of behaviors including sleep, anxiety, depression, and fear, to name but a few (Rolls et al., 2011; Kim et al., 2013; Tye et al., 2013; Courtin et al., 2014). More specifically, midbrain DA neurons and their striatal projections have also been selectively targeted resulting in behavioral modifications of food intake, cocaine consumption, conditioned place preference and aversion (by inhibition of DA activity via GABAergic VTA cells) (Tsai et al., 2009; Lobo et al., 2010; Domingos et al., 2011; Tan et al., 2012).

Optogenetic targeting of midbrain DA cells and their striatal projections, has also revealed interesting observations regarding their causal role in reward prediction, and possibly, behavioral flexibility. With regards to the causal role of DA in reward prediction (Kim et al., 2012), the authors showed that phasic activation of VTA DA neurons after a nose poke could drive operant responses in the absence of food reward. In another laboratory, a blocking procedure was used to demonstrate that activation of DA neurons at the time of reward delivery during compound stimulus presentation could artificially produce a conditioned response to the normally blocked cue. In other words, phasic DA stimulation at a point in time (reward delivery) when this would normally be absent could unblock learning (Steinberg et al., 2013).

In a separate study looking at manipulation of the GABAergic cells of the VTA on reward learning and its effect on DA release, optogenetic stimulation of VTA GABAergic neurons disrupted consummatory behavior but not if the VTA GABA projections to the NAc were targeted. Moreover, stimulation of the GABA neurons suppressed VTA DA firing and release in the NAc (Van Zessen et al., 2012). In a further study to characterize the VTA GABA projections to the NAc, it was found that activation of this pathway selectively inhibited cholinergic neurons of the NAc which in turn increased associative learning of an aversive predictive cue (Brown et al., 2012). Importantly, this effect was dopamine independent, as stimulation of GABA terminals in the NAc did not change baseline firing of VTA DA cells. Taken together, these studies confirm that within the VTA, DA activity regulates aspects related to appetitive reward learning. Moreover, these data highlight how the encoding of an aversive outcome may not only be signaled by DA cells projecting to the NAc but also by activation of cholinergic cells in the NAc that receive preferential input from VTA GABA neurons, extending the results from previous investigations (Tan et al., 2012).

With regards to the causal role of DA in behavioral flexibility, in a recent study (Adamantidis et al., 2011), the authors targeted the dopaminergic neurons of the VTA by injecting channelrhodopsin-2 (ChR2) in *Th-Cre* mice. The initial behavioral paradigm required mice to bar press one of two levers. The “active” lever resulted in food delivery plus optogenetic stimulation whereas bar pressing on the “inactive” lever resulted in the delivery of food only. Compared to controls (YFP mice), phasic DA stimulation enhanced the effects of food-reward seeking (i.e., mice bar pressed the active lever preferentially over the inactive). Interestingly, they also found that after a series of extinction sessions during which no food reward or phasic DA stimulation occurred, preferential lever pressing (to the initial active lever) could be reestablished by DA stimulation in the absence of both external cues and, critically, food reward. Finally, the authors used a reversal learning session where the relationship between the active (optical stimulation + no food reward) and inactive (no optical stimulation + no food reward) levers were switched, and demonstrated that ChR2 mice switched their lever pressing to the previously inactive lever compared to control mice. This finding is particularly important because it suggests that not only is the phasic DA signal driving and enhancing simple stimulus-reward associations but it is also causally involved in flexible behavioral adaptations that occur as a result of changes in stimulus-reward contingencies.

Behavioral flexibility has also been tested by optogenetic manipulations of dopamine receiving NAc neurons. In a recent study, dopamine D1 and D2 receptors were selectively targeted while *D1-cre* and *D2–cre* mice were performing a probabilistic switching task (Tai et al., 2012). The results showed that activation of D1 and D2 neurons was effective at increasing lose-shift behavior (i.e., moving from an incorrect to a correct response) compared to controls but had no effect on win-stay performance (i.e., repeating the previously rewarded response). Moreover, the effect was dependent on whether stimulation occurred before movement initiation but not if it was delayed by 150 ms. Interestingly, we recently found (Aquili et al., 2014) that non-specific optogenetic inhibition and not excitation of NAc shell neurons increased lose-shift behavior but only if the inhibition occurred during feedback of results (between lever pressing and rewards or non-rewards) but not during action selection (preceding a lever press). We speculated that inhibition of NAc cells during specific time segments may have weakened reward expectancy signals which would in turn facilitate switching to a correct response after an error.

Differential effects between NAc core and shell on learning have been observed using fast-scan cyclic voltammetry which may explain the contradictory findings from the two previous optogenetic studies. In fact, in one study cue-evoked dopamine release was larger and longer lasting in the NAc shell than in the core during goal-directed behavior for sucrose (Cacciapaglia et al., 2012). In two related studies, it was also found that concentrations of cue-evoked DA release closely tracked differences in reward magnitude in the NAc shell (Beyene et al., 2010) and reward delays in both NAc core and shell (Wanat et al., 2010). DA reward prediction error signals in the NAc core have also been reported using voltammetry (Hart et al., 2014). Here, using a probabilistic decision-making task, the authors found that dopamine concentrations varied systematically as differing degrees of reward uncertainty were introduced, in a manner closely resembling the predictions of reinforcement learning models and electrophysiological data of VTA DA neurons. Similarly, the observation that the DA phasic response to rewards gradually shifts to the earliest predictor of reinforcement over the course of learning as predicted by temporal difference models (Sutton and Barto, 1981) and validated by DA electrophysiological recordings, has been confirmed by voltammetric data (Sunsay and Rebec, 2008). These findings are important because changes in firing rates may not always reflect changes in DA release (Youngren et al., 1993), and these voltammetric data allow us to better establish the causal role of DA in reward learning.

Data from pharmacological manipulation of (mostly) dopamine D1 and D2 function in the striatum is another important component to take into account when trying to establish a causal link between neural activity and behavior. Dopamine depletion, for example, in the dorsomedial striatum results in reversal learning impairments (O'Neill and Brown, 2007). Moreover, in stimulant dependent individuals who display perseverative behaviors following an incorrect response during a reversal learning task, administration of a dopamine D2/3 antagonist reduced perseverative errors and improved caudate nucleus function (Ersche et al., 2011), and in separate study, administration of a D2 antagonist enhanced reward related prediction error signals in the striatum (Jocham et al., 2011). Conversely, stimulation of D2 (but not D1) receptors using the agonist quinpirole impaired goal-directed behavior and decision making (St Onge et al., 2011; Naneix et al., 2013) and broad inactivation of caudate nucleus cells disrupted the ability for flexible responses based on previous reward history (Muranishi et al., 2011). Interestingly, in monkeys, D2 receptor availability in the dorsal striatum was correlated with the number of reversal learning errors (Groman et al., 2011). Overall, these data suggest that abnormal increases/decreases in striatum DA activity via D1/D2 receptors causally influence several important measures of behavioral flexibility.

Studies that have looked at increasing dopamine concentration have demonstrated that DA stimulation by injection of amphetamine in the NAc core or shell increased instrumental responding to a conditioned stimulus predictive of reward (Pecina and Berridge, 2013), and administration of the dopamine precursor L-DOPA in older adults restored reward prediction error signaling (Chowdhury et al., 2013).

In conclusion, increasing evidence from optogenetic, voltammetry, and pharmacological studies over the recent years have added a new dimension to the established but mostly correlation role between the midbrain DA neurons and reward learning. This evidence suggests that this phasic response may have a causal role not only in reward prediction error signaling, but also in driving flexible behavioral adaptations to changes in stimulus-reward contingencies.

## Conflict of interest statement

The author declares that the research was conducted in the absence of any commercial or financial relationships that could be construed as a potential conflict of interest.

## References

[B1] Adamantidis A. R. Tsai H. C. Boutrel B. Zhang F. Stuber G. D. Budygin E. A. (2011). Optogenetic interrogation of dopaminergic modulation of the multiple phases of reward-seeking behavior. J. Neurosci. 31, 10829–10835 10.1523/JNEUROSCI.2246-11.2011 21795535 PMC3171183

[B2] Aquili L. Liu A. W. Shindou M. Shindou T. Wickens J. R. (2014). Behavioral flexibility is increased by optogenetic inhibition of neurons in the nucleus accumbens shell during specific time segments. Learn. Mem. 21, 223–231 10.1101/lm.034199.113 24639489 PMC3966536

[B3] Berridge K. C. Robinson T. E. Aldridge J. W. (2009). Dissecting components of reward: ‘liking’, ‘wanting’, and learning. Curr. Opin. Pharmacol. 9, 65–73 10.1016/j.coph.2008.12.014 19162544 PMC2756052

[B4] Beyene M. Carelli R. M. Wightman R. M. (2010). Cue-evoked dopamine release in the nucleus accumbens shell tracks reinforcer magnitude during intracranial self-stimulation. Neuroscience 169, 1682–1688 10.1016/j.neuroscience.2010.06.047 20600644 PMC2918713

[B5] Bromberg-Martin E. S. Matsumoto M. Hikosaka O. (2010a). Dopamine in motivational control: rewarding, aversive, and alerting. Neuron 68, 815–834 10.1016/j.neuron.2010.11.022 21144997 PMC3032992

[B6] Bromberg-Martin E. S. Matsumoto M. Hong S. Hikosaka O. (2010b). A pallidus-habenula-dopamine pathway signals inferred stimulus values. J. Neurophysiol. 104, 1068–1076 10.1152/jn.00158.2010 20538770 PMC2934919

[B7] Brooks A. M. Berns G. S. (2013). Aversive stimuli and loss in the mesocorticolimbic dopamine system. Trends Cogn. Sci. 17, 281–286 10.1016/j.tics.2013.04.001 23623264

[B8] Brown M. T. Tan K. R. O'Connor E. C. Nikonenko I. Muller D. Luscher C. (2012). Ventral tegmental area GABA projections pause accumbal cholinergic interneurons to enhance associative learning. Nature 492, 452–456 10.1038/nature11657 23178810

[B9] Cacciapaglia F. Saddoris M. P. Wightman R. M. Carelli R. M. (2012). Differential dopamine release dynamics in the nucleus accumbens core and shell track distinct aspects of goal-directed behavior for sucrose. Neuropharmacology 62, 2050–2056 10.1016/j.neuropharm.2011.12.027 22261383 PMC3433749

[B10] Chowdhury R. Guitart-Masip M. Lambert C. Dayan P. Huys Q. Duzel E. (2013). Dopamine restores reward prediction errors in old age. Nat. Neurosci. 16, 648–653 10.1038/nn.3364 23525044 PMC3672991

[B11] Cohen J. Y. Haesler S. Vong L. Lowell B. B. Uchida N. (2012). Neuron-type-specific signals for reward and punishment in the ventral tegmental area. Nature 482, 85–88 10.1038/nature10754 22258508 PMC3271183

[B12] Courtin J. Chaudun F. Rozeske R. R. Karalis N. Gonzalez-Campo C. Wurtz H. (2014). Prefrontal parvalbumin interneurons shape neuronal activity to drive fear expression. Nature 505, 92–96 10.1038/nature12755 24256726

[B13] Dayan P. Balleine B. W. (2002). Reward, motivation, and reinforcement learning. Neuron 36, 285–298 10.1016/S0896-6273(02)00963-7 12383782

[B14] Domingos A. I. Vaynshteyn J. Voss H. U. Ren X. Gradinaru V. Zang F. (2011). Leptin regulates the reward value of nutrient. Nat. Neurosci. 14, 1562–1568 10.1038/nn.2977 22081158 PMC4238286

[B15] Ersche K. D. Roiser J. P. Abbott S. Craig K. J. Muller U. Suckling J. (2011). Response perseveration in stimulant dependence is associated with striatal dysfunction and can be ameliorated by a D(2/3) receptor agonist. Biol. Psychiatry 70, 754–762 10.1016/j.biopsych.2011.06.033 21967987

[B16] Fiorillo C. D. Newsome W. T. Schultz W. (2008). The temporal precision of reward prediction in dopamine neurons. Nat. Neurosci. 11, 966–973 10.1038/nn.2159 18660807

[B17] Fiorillo C. D. Tobler P. N. Schultz W. (2003). Discrete coding of reward probability and uncertainty by dopamine neurons. Science 299, 1898–1902 10.1126/science.1077349 12649484

[B18] Fiorillo C. D. Yun S. R. Song M. R. (2013). Diversity and homogeneity in responses of midbrain dopamine neurons. J. Neurosci. 33, 4693–4709 10.1523/JNEUROSCI.3886-12.2013 23486943 PMC3873403

[B19] Groman S. M. Lee B. London E. D. Mandelkern M. A. James A. S. Feiler K. (2011). Dorsal striatal D2-like receptor availability covaries with sensitivity to positive reinforcement during discrimination learning. J. Neurosci. 31, 7291–7299 10.1523/JNEUROSCI.0363-11.2011 21593313 PMC3114883

[B20] Hart A. S. Rutledge R. B. Glimcher P. W. Phillips P. E. (2014). Phasic dopamine release in the rat nucleus accumbens symmetrically encodes a reward prediction error term. J. Neurosci. 34, 698–704 10.1523/JNEUROSCI.2489-13.2014 24431428 PMC3891951

[B21] Horvitz J. C. Stewart T. Jacobs B. L. (1997). Burst activity of ventral tegmental dopamine neurons is elicited by sensory stimuli in the awake cat. Brain Res. 759, 251–258 10.1016/S0006-8993(97)00265-5 9221945

[B22] Ilango A. Shumake J. Wetzel W. Scheich H. Ohl F. W. (2012). The role of dopamine in the context of aversive stimuli with particular reference to acoustically signaled avoidance learning. Front. Neurosci. 6:132 10.3389/fnins.2012.00132 23049495 PMC3442182

[B23] Jocham G. Klein T. A. Ullsperger M. (2011). Dopamine-mediated reinforcement learning signals in the striatum and ventromedial prefrontal cortex underlie value-based choices. J. Neurosci. 31, 1606–1613 10.1523/JNEUROSCI.3904-10.2011 21289169 PMC6623749

[B24] Joel D. Niv Y. Ruppin E. (2002). Actor-critic models of the basal ganglia: new anatomical and computational perspectives. Neural Netw. 15, 535–547 10.1016/S0893-6080(02)00047-3 12371510

[B25] Kim K. M. Baratta M. V. Yang A. Lee D. Boyden E. S. Fiorillo C. D. (2012). Optogenetic mimicry of the transient activation of dopamine neurons by natural reward is sufficient for operant reinforcement. PLoS ONE 7:e33612 10.1371/journal.pone.0033612 22506004 PMC3323614

[B26] Kim S. Y. Adhikari A. Lee S. Y. Marshel J. H. Kim C. K. Mallory C. S. (2013). Diverging neural pathways assemble a behavioural state from separable features in anxiety. Nature 496, 219–223 10.1038/nature12018 23515158 PMC6690364

[B27] Lobo M. K. Covington H. E. 3rd. Chaudhury D. Friedman A. K. Sun H. Damez-Werno D. (2010). Cell type-specific loss of BDNF signaling mimics optogenetic control of cocaine reward. Science 330, 385–390 10.1126/science.1188472 20947769 PMC3011229

[B28] Matsumoto M. Hikosaka O. (2009). Two types of dopamine neuron distinctly convey positive and negative motivational signals. Nature 459, 837–841 10.1038/nature08028 19448610 PMC2739096

[B29] May P. J. McHaffie J. G. Stanford T. R. Jiang H. Costello M. G. Coizet V. (2009). Tectonigral projections in the primate: a pathway for pre-attentive sensory input to midbrain dopaminergic neurons. Eur. J. Neurosci. 29, 575–587 10.1111/j.1460-9568.2008.06596.x 19175405 PMC2856337

[B30] McClure S. M. Daw N. D. Montague P. R. (2003). A computational substrate for incentive salience. Trends Neurosci. 26, 423–428 10.1016/S0166-2236(03)00177-2 12900173

[B31] Mirenowicz J. Schultz W. (1994). Importance of unpredictability for reward responses in primate dopamine neurons. J. Neurophysiol. 72, 1024–1027 7983508 10.1152/jn.1994.72.2.1024

[B32] Montague P. R. Dayan P. Sejnowski T. J. (1996). A framework for mesencephalic dopamine systems based on predictive Hebbian learning. J. Neurosci. 16, 1936–1947 8774460 10.1523/JNEUROSCI.16-05-01936.1996 PMC6578666

[B33] Montague P. R. Hyman S. E. Cohen J. D. (2004). Computational roles for dopamine in behavioural control. Nature 431, 760–767 10.1038/nature03015 15483596

[B34] Morita K. Morishima M. Sakai K. Kawaguchi Y. (2013). Dopaminergic control of motivation and reinforcement learning: a closed-circuit account for reward-oriented behavior. J. Neurosci. 33, 8866–8890 10.1523/JNEUROSCI.4614-12.2013 23678129 PMC6618820

[B35] Morris G. Nevet A. Arkadir D. Vaadia E. Bergman H. (2006). Midbrain dopamine neurons encode decisions for future action. Nat. Neurosci. 9, 1057–1063 10.1038/nn1743 16862149

[B36] Muranishi M. Inokawa H. Yamada H. Ueda Y. Matsumoto N. Nakagawa M. (2011). Inactivation of the putamen selectively impairs reward history-based action selection. Exp. Brain Res. 209, 235–246 10.1007/s00221-011-2545-y 21298425 PMC3041916

[B37] Naneix F. Marchand A. R. Pichon A. Pape J. R. Coutureau E. (2013). Adolescent stimulation of D2 receptors alters the maturation of dopamine-dependent goal-directed behavior. Neuropsychopharmacology 38, 1566–1574 10.1038/npp.2013.55 23443719 PMC3682151

[B38] Niv Y. (2007). Cost, benefit, tonic, phasic: what do response rates tell us about dopamine and motivation? Ann. N.Y. Acad. Sci. 1104, 357–376 10.1196/annals.1390.018 17416928

[B39] Nomoto K. Schultz W. Watanabe T. Sakagami M. (2010). Temporally extended dopamine responses to perceptually demanding reward-predictive stimuli. J. Neurosci. 30, 10692–10702 10.1523/JNEUROSCI.4828-09.2010 20702700 PMC3297489

[B40] O'Doherty J. P. Buchanan T. W. Seymour B. Dolan R. J. (2006). Predictive neural coding of reward preference involves dissociable responses in human ventral midbrain and ventral striatum. Neuron 49, 157–166 10.1016/j.neuron.2005.11.014 16387647

[B41] O'Neill M. Brown V. J. (2007). The effect of striatal dopamine depletion and the adenosine A2A antagonist KW-6002 on reversal learning in rats. Neurobiol. Learn. Mem. 88, 75–81 10.1016/j.nlm.2007.03.003 17467309

[B42] Pan W. X. Schmidt R. Wickens J. R. Hyland B. I. (2008). Tripartite mechanism of extinction suggested by dopamine neuron activity and temporal difference model. J. Neurosci. 28, 9619–9631 10.1523/JNEUROSCI.0255-08.2008 18815248 PMC6671219

[B43] Pecina S. Berridge K. C. (2013). Dopamine or opioid stimulation of nucleus accumbens similarly amplify cue-triggered ‘wanting’ for reward: entire core and medial shell mapped as substrates for PIT enhancement. Eur. J. Neurosci. 37, 1529–1540 10.1111/ejn.12174 23495790 PMC4028374

[B44] Redgrave P. Gurney K. (2006). The short-latency dopamine signal: a role in discovering novel actions? Nat. Rev. Neurosci. 7, 967–975 10.1038/nrn2022 17115078

[B45] Redgrave P. Gurney K. Reynolds J. (2008). What is reinforced by phasic dopamine signals? Brain Res. Rev. 58, 322–339 10.1016/j.brainresrev.2007.10.007 18055018

[B46] Roesch M. R. Calu D. J. Schoenbaum G. (2007). Dopamine neurons encode the better option in rats deciding between differently delayed or sized rewards. Nat. Neurosci. 10, 1615–1624 10.1038/nn2013 18026098 PMC2562672

[B47] Rolls A. Colas D. Adamantidis A. Carter M. Lanre-Amos T. Heller H. C. (2011). Optogenetic disruption of sleep continuity impairs memory consolidation. Proc. Natl. Acad. Sci. U.S.A. 108, 13305–13310 10.1073/pnas.1015633108 21788501 PMC3156195

[B48] Schultz W. (1998). Predictive reward signal of dopamine neurons. J. Neurophysiol. 80, 1–27 9658025 10.1152/jn.1998.80.1.1

[B49] Schultz W. (2002). Getting formal with dopamine and reward. Neuron 36, 241–263 10.1016/S0896-6273(02)00967-4 12383780

[B50] Schultz W. (2010). Multiple functions of dopamine neurons. F1000 Biol. Rep. 2:2 10.3410/B2-2 20948813 PMC2948345

[B51] Schultz W. Dayan P. Montague P. R. (1997). A neural substrate of prediction and reward. Science 275, 1593–1599 10.1126/science.275.5306.1593 9054347

[B52] Steinberg E. E. Keiflin R. Boivin J. R. Witten I. B. Deisseroth K. Janak P. H. (2013). A causal link between prediction errors, dopamine neurons and learning. Nat. Neurosci. 16, 966–973 10.1038/nn.3413 23708143 PMC3705924

[B53] St Onge J. R. Abhari H. Floresco S. B. (2011). Dissociable contributions by prefrontal D1 and D2 receptors to risk-based decision making. J. Neurosci. 31, 8625–8633 10.1523/JNEUROSCI.1020-11.2011 21653866 PMC6623317

[B54] Sunsay C. Rebec G. V. (2008). Real-time dopamine efflux in the nucleus accumbens core during Pavlovian conditioning. Behav. Neurosci. 122, 358–367 10.1037/0735-7044.122.2.358 18410174 PMC2664557

[B55] Sutton R. S. Barto A. G. (1981). Toward a modern theory of adaptive networks: expectation and prediction. Psychol. Rev. 88, 135–170 10.1037/0033-295X.88.2.135 7291377

[B56] Tai L. H. Lee A. M. Benavidez N. Bonci A. Wilbrecht L. (2012). Transient stimulation of distinct subpopulations of striatal neurons mimics changes in action value. Nat. Neurosci. 15, 1281–1289 10.1038/nn.3188 22902719 PMC3951287

[B57] Takahashi Y. K. Roesch M. R. Stalnaker T. A. Haney R. Z. Calu D. J. Taylor A. R. (2009). The orbitofrontal cortex and ventral tegmental area are necessary for learning from unexpected outcomes. Neuron 62, 269–280 10.1016/j.neuron.2009.03.005 19409271 PMC2693075

[B58] Tan K. R. Yvon C. Turiault M. Mirzabekov J. J. Doehner J. Labouebe G. (2012). GABA neurons of the VTA drive conditioned place aversion. Neuron 73, 1173–1183 10.1016/j.neuron.2012.02.015 22445344 PMC6690362

[B59] Thirkettle M. Walton T. Shah A. Gurney K. Redgrave P. Stafford T. (2013). The path to learning: action acquisition is impaired when visual reinforcement signals must first access cortex. Behav. Brain Res. 243, 267–272 10.1016/j.bbr.2013.01.023 23380676

[B60] Tsai H. C. Zhang F. Adamantidis A. Stuber G. D. Bonci A. De Lecea L. (2009). Phasic firing in dopaminergic neurons is sufficient for behavioral conditioning. Science 324, 1080–1084 10.1126/science.1168878 19389999 PMC5262197

[B61] Tye K. M. Mirzabekov J. J. Warden M. R. Ferenczi E. A. Tsai H. C. Finkelstein J. (2013). Dopamine neurons modulate neural encoding and expression of depression-related behaviour. Nature 493, 537–541 10.1038/nature11740 23235822 PMC4160519

[B62] Van Zessen R. Phillips J. L. Budygin E. A. Stuber G. D. (2012). Activation of VTA GABA neurons disrupts reward consumption. Neuron 73, 1184–1194 10.1016/j.neuron.2012.02.016 22445345 PMC3314244

[B63] Wanat M. J. Kuhnen C. M. Phillips P. E. (2010). Delays conferred by escalating costs modulate dopamine release to rewards but not their predictors. J. Neurosci. 30, 12020–12027 10.1523/JNEUROSCI.2691-10.2010 20826665 PMC2946195

[B64] Wassum K. M. Ostlund S. B. Balleine B. W. Maidment N. T. (2011). Differential dependence of Pavlovian incentive motivation and instrumental incentive learning processes on dopamine signaling. Learn. Mem. 18, 475–483 10.1101/lm.2229311 21693635 PMC3125614

[B65] Wassum K. M. Ostlund S. B. Loewinger G. C. Maidment N. T. (2013). Phasic mesolimbic dopamine release tracks reward seeking during expression of pavlovian-to-instrumental transfer. Biol. Psychiatry 73, 747–755 10.1016/j.biopsych.2012.12.005 23374641 PMC3615104

[B66] Youngren K. D. Daly D. A. Moghaddam B. (1993). Distinct actions of endogenous excitatory amino acids on the outflow of dopamine in the nucleus accumbens. J. Pharmacol. Exp. Ther. 264, 289–293 8093728

[B67] Zhang J. Berridge K. C. Tindell A. J. Smith K. S. Aldridge J. W. (2009). A neural computational model of incentive salience. PLoS Comput. Biol. 5:e1000437 10.1371/journal.pcbi.1000437 19609350 PMC2703828

